# Accumulation of airborne microplastics in lichens from a landfill dumping site (Italy)

**DOI:** 10.1038/s41598-021-84251-4

**Published:** 2021-02-25

**Authors:** Stefano Loppi, Brett Roblin, Luca Paoli, Julian Aherne

**Affiliations:** 1 University of Siena, Siena, Italy; 2 School of the Environment, Trent University, Peterborough, Canada; 3 University of Pisa, Pisa, Italy

**Keywords:** Environmental impact, Environmental sciences, Atmospheric chemistry, Environmental monitoring

## Abstract

The aim of this study was to assess if lichens (*Flavoparmelia caperata*) surrounding a landfill dumping site in Italy accumulated higher amounts of microplastics compared with lichens at more distant sites. Lichen samples were collected at three sites along a transect from the landfill: close (directly facing the landfill), intermediate (200 m), and remote (1500 m). Anthropogenic microparticles (fibres and fragments) were determined visually after wet peroxide digestion of the samples, and microplastics were identified based on a hot needle test; the type of plastic was identified by micro-Raman analysis. The results showed that lichens collected in the vicinity of the landfill accumulated the highest number of anthropogenic microfibres and fragments (147 mp/g dw), and consequently microplastics (79 mp/g dw), suggesting that the impact of landfill emissions is spatially limited. The proportion of fibres and fragments identified as microplastics was 40% across all sites and the most abundant polymer type was polyester or polyethylene terephthalate (68%). These results clearly indicated that lichens can effectively be used to monitor the deposition of microplastics.

## Introduction

The term microplastic is relatively new^1^ and refers to small pieces of waste plastic less than 5 mm in length^2^, which have been widely observed in the environment as a consequence of plastic pollution. Microplastics are classified as either primary or secondary; the former directly emitted to the environment, and the latter indirectly through the breakdown of larger plastics^3^. Further, based on shape, microplastics are classified as microfibres (with one longer dimension) or microfragments and microfilms (without a dominant dimension).

One of the main features of plastic is its durability and persistence, leading to its accumulation in the environment and consequent worldwide concern^4^. Although marine and freshwater environments have widely been investigated for microplastic pollution, it is now clear that the atmosphere is also largely polluted by microplastics^3,5^, even at remote sites^6,7^.

The management of waste plastic should ensure that recycling, burning in combustion facilities, or burying in landfills, is carried out in such a way that plastic is reused, destroyed or contained, and not released into the wider environment. However, during dumping at landfills, microplastics can be produced and become airborne as a consequence of operations and wind-blown littering^8^.

Similar to the monitoring of other important persistent air pollutants, such as trace metals, living organisms (biomonitors) may be very useful for investigating the atmospheric deposition of microplastics, but to date, studies on this topic are very scant. Only one study has evaluated the use of bryophytes (*Hylocomium splendens*) as a biomonitor for airborne anthropogenic microfibres^9^. To the best of our knowledge, this is the first paper exploring the possibility of using lichens as biomonitors for airborne microplastics. The aim of this study was to assess if lichens, collected from around a landfill dumping site in Italy, accumulated higher amounts of microplastics compared with lichens at more removed (distant) sites.

## Results

Anthropogenic fibres and fragments were observed in all samples (n = 9); replicate samples along three transects from the landfill, i.e., close (directly facing), intermediate (200 m), and remote (1500 m). In total 438 particles were observed across all sampling locations, ranging from 321 (total particles at triplicate close sites) to 55 (remote) particles (see Supporting Information Table SI-1 and Figure SI-1). Lichens at sites close to the landfill accumulated the highest concentration (number per dry weight of lichen) of anthropogenic microfibres and fragments (60 and 86 per g dw, respectively), while values at the intermediate and remote sites were similar and much lower (Table 1), especially for fragments (e.g., 8 per g dw at the remote site). The percentage of microfibres was similar at sites close and intermediate (41%), and much higher at the remote site (73%; Table 1). The median lengths of microfibres (550–796 µm) and fragments (45–66 µm) were comparable across sites (Table 1). However, maximum fibre length decreased with distance from the landfill, ranging from 4021 to 3212 µm at close–remote sites (see Supporting Information Table SI-1 and Figure SI-2).

**Table 1:** Median values (95% Confidence Interval) of anthropogenic fragments and fibres, and microplastics accumulated by the lichen *Flavoparmelia caperata* at sites located at distance from the landfill.

	Close (facing)	Intermediate (200 m)	Remote (1500 m)
Fragments (nr/g dw)	86 (0–101)^a^	18 (0–20)^b^	8 (0–9)^b^
Fibres (nr/g dw)	60 (0–72)^a^	19 (17–25)^b^	23 (22–28)^b^
Fibres and fragments (nr/g dw)	147 (0–175)^a^	35 (0–39)^b^	31 (13–37)^b^
Microplastics (nr/g dw)^†^	79 (0–95)^a^	13 (0–15)^b^	7 (3–9)^b^
Fibres (%)	41 (35–43)^a^	41 (29–57)^a^	73 (70–99)^b^
Fragment length (µm)	45 (37–51)	50 (27–55)	66 (37–90)
Fibre length (µm)	634 (500–714)	550 (112–791)	796 (376–1043)

Different letters indicate statistically significant (P < 0.05) differences.

^†^Estimated using the proportion of fibres and fragments that were identified as plastic, 54% at close sites, 38% at intermediate, and 23% at remote sites.

The proportion of anthropogenic fibres and fragments identified as microplastics (synthetic petrochemical based polymers) was 40% across all sites (equally split between fibres and fragments), ranging from 54% at the close sites, 38% at the intermediate sites, and 23% at the remote sites. The remaining fibres and fragments (60%) were classified as being synthetic organic, likely cellulose, e.g., cotton, wool, or regenerated cellulose.

The most abundant polymer type was polyester or polyethylene terephthalate (PET) at 68%, followed by polyethylene at 26%, and polystyrene at 5% (see Supporting Information Figure SI-3).

## Discussion

The results of the present study are fully consistent with those of Paoli et al.^10^. These Authors investigated the diversity of epiphytic lichens, the bioaccumulation of trace elements by *F. caperata*, and the ecophysiological status of this lichen at the same study sites. They found that the index of lichen diversity was the lowest, and the content of heavy metals (Cr, Cd, Cu, Fe, Ni, and Zn) was the highest, close to the landfill. Clear stress signals were observed in lichens growing close to the facility, i.e., discoloration, necrosis, membrane lipid peroxidation, lower ergosterol content, higher dehydrogenase activity, and decreased photosynthetic efficiency. As a consequence, the present results fully support the suggestion that analysis of airborne microplastics accumulated by lichens is a suitable tool to identify areas differentially impacted by the landfill. In addition, the results also confirm that landfills are an important source of airborne microplastics. While this result is somewhat common sense, this is the first study to assess airborne microplastics from a landfill.

In comparison to the data reported by Roblin and Aherne^9^ for moss samples from background regions in Ireland, the only other study that investigated the use of living organisms to monitor the deposition of airborne microplastics, our study showed similar values of accumulated microfibres (15–30 mf/g dw). However, taking the exposure time into account, our lichens are roughly representative of one-year growth, while the moss samples analysed by Roblin and Aherne^9^ were considered to represent 2–3 years of growth. Hence, the lichen values (19–60 mf/g dw) may be up to six times higher than the moss values. In addition, our unpublished data showed that the weight of the specific surface area of the lichen *F. caperata* (50 g/m^2^) is ca. three times higher than that of the moss *Hylocomium splendens* (15 g/m^2^) sampled by Roblin and Aherne^9^, leading to estimated values in lichens up to 20 times higher than in moss. These estimates are consistent with the fact that the moss samples were taken from remote locations distant for anthropogenic sources, while lichens were collected from sites around a landfill. Nevertheless, Dehghani et al.^11^ reported microplastics in street dust of Tehran in the range 3–18 mp/g, which, assuming a tenfold higher specific weight of dust leads to 30–180 mp/g, which is fully comparable with our median range of 31–147 mp/g dw.

It should be noted that in this study an epiphytic (tree inhabiting) lichen species was sampled and analysed. This may have several implications. Firstly, lichen thalli were taken from tree trunks, and hence were overall vertically exposed, and as a consequence may have reduced ability to intercept the deposition of microplastics, compared with horizontally exposed moss cushions over a generally flat soil surface. Secondly, tree canopies may have a two-fold effect on microplastic deposition: interception and sequestration, and possible subsequent release following a precipitation event; as a consequence, accumulation of microplastics by epiphytic lichens may be either diminished by the canopy sheltering effect or enhanced by microplastics being washed down along the trunk in the stemflow. Interestingly, Klein and Fisher^12^ reported a higher deposition of microplastics under Douglas fir, which was attributed to enhanced atmospheric scavenging by their evergreen canopy, compared with under the canopy of deciduous beech trees or in open space, the latter experiencing similar values. In the present study, lichens were collected on the trunks of deciduous oak trees.

The relationship of microplastic deposition with precipitation is controversial. Allen et al.^6^ suggested that a link with precipitation, either in the form of rainfall or snow, does exist. This conclusion is supported by Bergman et al.^7^, who found microplastics accumulated in snow cores from the Alps and the Arctic. Similarly, Dris et al.^5^ found that precipitation events may be important drivers of atmospheric deposition of microplastics. However, Klein and Fisher^12^ failed to find any correlation between the deposition of microplastics and the amount of precipitation. Wright et al.^13^ suggested that meteorological parameters may be important for remote areas away from sources, but less important for urban areas that are sources of emission. Further, depending on regional climate, Brahney et al.^14^ observed that dry deposition of microplastics was generally equal if not greater than wet deposition in the western US. In the current study, it is likely that the accumulation of airborne microplastics by dry deposition is dominant owing to the much lower rainfall input in a Mediterranean-type climate like that of Italy compared with other European sites as quoted above, i.e., Pyrenees^6^, Alps^7^, Paris^5^, Hamburg^12^, and London^13^. It is important to note that in the current study, all sites were equally exposed to background microplastic deposition; however, sites closest to the landfill experienced higher deposition owing to their proximity to the emissions source.

The dominant shape, fibre or fragment, reported in previous studies has a wide array of variability: urban studies at Paris^5^, Dongguan^15^, and London^13^ reported a much higher proportion of microfibres (around 90–95%) compared to fragments. In contrast, studies at Hamburg^12^, Pyrenees^6^, Shanghai^15^, and Tehran^11^ found that fragments were dominant (57–95%) compared to fibres. Our results showed variable proportions of microfibres, ranging from 41% at sites up to 200 m from the landfill (close sites), to 73% at more removed (1500 m) sites. Nonetheless, consistent with the above studies, median lengths were in the range 45–66 µm for microfragments and 550–796 µm for microfibres. The most abundant polymer type at the study sites was polyester or PET, which was similar to studies in Paris, Hamburg, and the Pyrenees.

There is no doubt that microplastics are subjected to long-range atmospheric transport^3,17^, but the extent of this phenomenon is still largely unknown. Our results clearly indicated that the investigated landfill is a source of airborne microplastics, irrespective of fibres or fragments, but also showed that the amount of microplastics decreased exponentially with distance, from 79 mp/g dw at the close sites, to 13 mp/g dw at 200 m, and to 7 mp/g dw at 1500 m from the landfill. This suggests that long-range transport in our study was limited. Nonetheless, the dominance of fibres at the remote sites suggests that fibres may be more predisposed to longer atmospheric suspension (or that fragments deposit closer to source). In this context, the potential role of trees and forest vegetation in protecting the surrounding environment from the spread of microplastics remains to be explored in detail and approached experimentally.

## Conclusions

This is the first study to investigate the use of lichens as bioaccumulators of airborne microplastics. In addition, it is the first study to investigate the quantitative contribution and spatial extent of landfills as a source of microplastics to the wider environment. The results show that lichens collected in the vicinity of the landfill clearly accumulated the highest number of anthropogenic microfibres and fragments, and consequently microplastics, and suggests that the impact is spatially limited. These results clearly indicated that besides the classical biomonitoring outputs, i.e., biodiversity counts, bioaccumulation of trace elements, and ecophysiological parameters, lichens can be profitably used also to monitor the deposition of microplastics and are valid bioindicators of environmental quality surrounding landfills.

## Materials and methods

### Study area

The investigated landfill (43° 52′ 52″ N, 10° 53′ 21″ E, ca. 60 m asl) is located in Tuscany, central Italy, and has been in operation since 1996, with a capacity of 420 tons/day. It extends over an area of 160,000 m^2^ with a volume of ca. 3 million m^3^. The disposed material consists of industrial non-hazardous wastes, and includes scraps of paper, plastics and metals, packaging, spent tires, textile products, building materials, ashes from municipal solid waste incinerators, polluted terrain from environmental reclamation, etc.

### Experimental design

Based on previous lichenological studies^10^, sampling units were classified into three groups according to their distance from the landfill: Group 1: close, sites directly facing the landfill; Group 2: intermediate, sites located at about 200 m from the landfill; and Group 3: remote, sites located at about 1500 m from the landfill. Group 1 corresponded to impacted sites, while group 3 to sites with negligible impact. For each group, three sampling sites (replicates) were investigated along three transects from the landfill. The transects were located S–SW of the landfill, aligned with the prevailing winds, which blow in this direction for ca. 200 days per year, with an average wind speed of about 7 m/s.

At each site, samples of the foliose lichen *Flavoparmelia caperata* were collected for the analysis of microplastics. The lichen species was chosen because of its wide distribution in the study area and because it has already been used to investigate the deposition of trace elements at the same landfill^10,18^. At each sampling site, 10–30 thalli growing on the bole of oak trees (*Quercus cerris* and *Q. pubescens*) were harvested from all cardinal exposures, at 1–2 m from ground. Only the peripheral part of the thalli (up to 5 mm from lobe tips) was selected for the analysis; in *F. caperata* this part roughly corresponds to the last year of growth and can be easily separated from the bark, being distinguishable by a paler colour and absence of rhizinae.

### Microplastic analysis

In the laboratory, air-dried (residual water < 10%) lichen samples were weighed, placed into glass beakers, and individually digested using a wet peroxide oxidation method^9^. Samples were then vacuum filtered onto glass-fibre filter papers (Fisherbrand G6 [09-804-42A]: 1.6 μm) and dyed with 1 mL of Rose Bengal (4,5,6,7-tetrachloro-2′,4′,5′,7′-tetraiodofluorescein, 200 mg/L) to help visually distinguish synthetic material from organic matter^9^. The dyed filter papers were transferred to petri dishes for storage and for assessment of microplastics.

The filter papers were analysed for the presence of microplastics using a stereomicroscope (Leica EZ4W with EZ4W0170 camera), following a five-criterium method^9^. Microfibres and fragments that met at least two of the criteria, and were not stained by Rose Bengal, were considered anthropogenic^19^. To check for possible contamination, procedural open-air blanks were also analysed following the same method as the lichen samples. Microfibres and fragments were photographed and then measured using the open source Image processing software ImageJ.

In general, the term microplastic is often used to refer to both plastic and non-plastic particles (fibres, films, foams, and fragments) that are less than 5 mm in length. Here we refer to synthetic cellulose (cotton, rayon, acetate, etc.) and petrochemical-based particles as anthropogenic fibres and fragments, and we refer to the proportion identified as petrochemical-based polymers only as microplastics. To determine the proportion of microplastics, a hot needle test was conducted on a randomly selected subset of anthropogenic microfibres and fragments (> 20%). Following Norén^20^ and Hidalgo-Ruz et al.^21^, a hot needle was pressed against the tip of each randomly selected fibre or fragment, and if the sample melted it was identified as plastic.

### Raman analysis

All particles identified as plastic were analyzed using micro-Raman spectroscopy (WITec, operated by WITec Control) to determine polymer composition. Fibres were analyzed using a 785 nm laser to limit particle analysis issues at 50 ×–100 × objectives and adjustable power (ranging from 10 to 40 mW), and fragments were analyzed using a 532 nm laser at 20 ×–50 × objectives and adjustable power (power ranged from 15 to 20 mW). Spectra were recorded in the wavenumber range of 0–1800 cm^–1^ for fibres and 0–3600 cm^–1^ for fragments. The spectra were analyzed through a commercial library (KnowItAll, Bio-Rad) to confirm polymer identity (see Supporting Information Figure SI-3).

### Statistical analysis

Owing to the limited dataset, a two-sided permutation test (P < 0.05) was used to check for differences in fibres, fragments, and microplastics between groups. Results are presented as medians along with their bootstrapped 95% confidence intervals (note that the confidence limits are not necessarily symmetric around the sample estimate as is the case when standard errors are used to construct the confidence intervals). All statistical analysis was carried out using the free software R^22^.

## Supplementary Information

Supplementary Information

## Data Availability

All data generated or analysed during this study are included in this published article (and its Supplementary Information files).
