# Supplementary material for: Accumulation of airborne microplastics in lichens from a landfill dumping site (Italy)
Source: Sci Rep. 2021 Feb 25;11:4564. doi: 10.1038/s41598-021-84251-4 (PMC7907242; doi:10.1038/s41598-021-84251-4)
Supplement: Supplementary file 1 — Supplementary Information [file 41598_2021_84251_MOESM1_ESM.doc]

**Supporting Information**

**Accumulation of airborne microplastics in lichens from a landfill dumping site (Italy)**

Stefano Loppi^1^, Brett Roblin^2^, Luca Paoli^3^, Julian Aherne^2^

*^1^University of Siena, Italy; ^2^School of the Environment, Trent University, Canada; ^3^University of Pisa, Italy*

Table SI-1. The length (mm) of each anthropogenic microfibre (n = 438) extracted from lichen collected from the three study sites varying in distance from a landfill (Close, Intermediate and Remote). Each site has three replicates (1, 2 and 3), the mass (g) of each replicated is also given.

|  | Close |  |  | Intermediate | |  | Remote |  |
| --- | --- | --- | --- | --- | --- | --- | --- | --- |
| C01* | C02 | C03 | I01 | I02 | I03 | R01 | R02 | R03 |
| 0.44 g | 0.42 g | 0.43 g | 0.55 g | 0.39 g | 0.43 g | 0.66 g | 0.44 g | 0.39 g |
| 0.289 | 0.063 | 0.121 | 0.112 | 0.473 | 0.129 | 1.608 | 0.468 | 0.367 |
| 0.844 | 0.546 | 0.189 | 0.277 | 2.029 | 1.686 | 0.672 | 0.233 | 3.212 |
| 1.262 | 0.247 | 0.35 | 1.812 | 0.55 | 0.782 | 0.548 | 1.775 | 3.071 |
| 1.503 | 0.554 | 0.17 | 0.309 | 0.114 | 0.256 | 2.25 | 1.843 | 0.84 |
| 0.365 | 3.152 | 0.313 | 1.079 | 0.222 | 0.598 | 0.464 | 0.661 | 0.402 |
| 0.415 | 0.396 | 0.235 | 0.334 | 0.744 | 0.988 | 1.111 | 0.337 | 2.911 |
| 0.422 | 0.505 | 0.98 | 1.745 | 2.302 | 0.499 | 0.772 | 2.944 | 1.116 |
| 0.804 | 0.517 | 1.652 | 0.038 | 3.69 | 0.164 | 0.28 | 0.622 | 1.052 |
| 0.977 | 0.677 | 1.813 | 0.045 | 0.05 | 0.033 | 0.338 | 0.05 | 0.93 |
| 0.643 | 1.723 | 1.705 | 0.049 | 0.13 | 0.029 | 0.235 | 0.036 | 0.145 |
| 0.255 | 0.127 | 0.478 | 0.05 | 0.046 | 0.138 | 0.552 | 0.066 | 0.089 |
| 0.543 | 0.211 | 0.329 | 0.042 | 0.119 | 0.215 | 1.591 |  | 0.081 |
| 0.567 | 0.318 | 0.948 | 0.098 | 0.108 | 0.039 | 0.819 |  |  |
| 1.522 | 0.572 | 1.026 | 0.04 | 0.127 | 0.057 | 0.35 |  |  |
| 0.563 | 0.431 | 0.343 | 0.234 | 0.035 | 0.03 | 1.571 |  |  |
| 0.091 | 0.405 | 0.837 | 0.158 | 0.024 |  | 0.167 |  |  |
| 0.157 | 1.036 | 0.299 | 0.078 | 0.084 |  | 0.076 |  |  |
| 0.836 | 0.86 | 0.2 |  | 0.028 |  | 0.049 |  |  |
| 0.363 | 1.047 | 0.258 |  | 0.084 |  | 0.037 |  |  |
| 0.333 | 0.553 | 2.746 |  | 0.103 |  | 0.095 |  |  |
| 0.155 | 0.061 | 1.881 |  | 0.039 |  | 0.035 |  |  |
| 0.49 | 0.045 | 4.021 |  | 0.064 |  | 0.039 |  |  |
| 0.335 | 0.025 | 1.083 |  | 0.048 |  | 0.024 |  |  |
| 0.803 | 0.107 | 3.29 |  | 0.031 |  | 0.033 |  |  |
| 1.722 | 0.015 | 3.23 |  | 0.055 |  | 0.035 |  |  |
| 1.623 | 0.032 | 0.377 |  | 0.073 |  | 0.234 |  |  |
| 0.373 | 0.034 | 0.038 |  | 0.04 |  | 0.168 |  |  |
| 0.371 | 0.033 | 0.068 |  | 0.048 |  | 0.165 |  |  |
| 1.593 | 0.068 | 0.042 |  | 0.05 |  | 0.042 |  |  |
| 0.643 | 0.091 | 0.054 |  | 0.048 |  | 0.108 |  |  |
| 0.768 | 0.035 | 0.026 |  |  |  | 0.043 |  |  |
| 0.298 | 0.032 | 0.028 |  |  |  | 0.174 |  |  |
| 0.175 | 0.11 | 0.048 |  |  |  |  |  |  |
| 0.952 | 0.023 | 0.064 |  |  |  |  |  |  |
| 0.968 | 0.093 | 0.039 |  |  |  |  |  |  |
| 0.395 | 0.131 | 0.06 |  |  |  |  |  |  |
| 0.711 | 0.037 | 0.108 |  |  |  |  |  |  |
| 0.517 | 0.051 | 0.103 |  |  |  |  |  |  |
| 0.622 | 0.08 | 0.04 |  |  |  |  |  |  |
| 0.289 | 0.047 | 0.126 |  |  |  |  |  |  |
| 0.316 | 0.031 | 0.04 |  |  |  |  |  |  |
| 0.441 | 0.025 | 0.047 |  |  |  |  |  |  |
| 0.634 | 0.047 | 0.054 |  |  |  |  |  |  |
| 2.41 | 0.03 | 0.028 |  |  |  |  |  |  |
| 0.325 | 0.09 | 0.183 |  |  |  |  |  |  |
| 0.709 | 0.045 | 0.24 |  |  |  |  |  |  |
| 0.608 | 0.03 | 0.036 |  |  |  |  |  |  |
| 0.676 | 0.031 | 0.095 |  |  |  |  |  |  |
| 1.221 | 0.032 | 0.036 |  |  |  |  |  |  |
| 0.65 | 0.035 | 0.061 |  |  |  |  |  |  |
| 0.93 |  | 0.041 |  |  |  |  |  |  |
| 0.981 |  | 0.069 |  |  |  |  |  |  |
| 0.151 |  | 0.097 |  |  |  |  |  |  |
| 1.503 |  | 0.058 |  |  |  |  |  |  |
| 0.634 |  | 0.018 |  |  |  |  |  |  |
| 1.669 |  | 0.056 |  |  |  |  |  |  |
| 0.189 |  | 0.041 |  |  |  |  |  |  |
| 0.525 |  | 0.204 |  |  |  |  |  |  |
| 0.631 |  | 0.035 |  |  |  |  |  |  |
| 0.743 |  | 0.033 |  |  |  |  |  |  |
| 0.571 |  | 0.037 |  |  |  |  |  |  |
| 1.173 |  | 0.053 |  |  |  |  |  |  |
| 0.563 |  | 0.031 |  |  |  |  |  |  |
| 0.541 |  |  |  |  |  |  |  |  |
| 0.905 |  |  |  |  |  |  |  |  |
| 1.424 |  |  |  |  |  |  |  |  |
| 0.638 |  | #1 |  |  |  |  |  |  |
| 1.692 |  | 0.075 |  |  |  |  |  |  |
| 2.339 |  | 0.055 |  |  |  |  |  |  |
| 0.976 |  | 0.211 |  |  |  |  |  |  |
| 3.73 |  | 0.28 |  |  |  |  |  |  |
| 1.12 |  | 0.028 |  |  |  |  |  |  |
| 2.056 |  | 0.045 |  |  |  |  |  |  |
| 0.975 |  | 0.116 |  |  |  |  |  |  |
| 0.841 |  | 0.068 |  |  |  |  |  |  |
| 0.862 |  | 0.036 |  |  |  |  |  |  |
| 2.168 |  | 0.023 |  |  |  |  |  |  |
| 0.185 |  | 0.069 |  |  |  |  |  |  |
| 1.047 |  | 0.016 |  |  |  |  |  |  |
| 0.393 |  | 0.039 |  |  |  |  |  |  |
| 0.8 |  | 0.079 |  |  |  |  |  |  |
| 1.566 |  | 0.142 |  |  |  |  |  |  |
| 0.56 |  | 0.187 |  |  |  |  |  |  |
| 1.027 |  | 0.07 |  |  |  |  |  |  |
| 0.405 |  | 0.056 |  |  |  |  |  |  |
| 0.671 |  | 0.041 |  |  |  |  |  |  |
| 0.685 |  | 0.024 |  |  |  |  |  |  |
| 0.134 |  | 0.013 |  |  |  |  |  |  |
| 0.222 |  | 0.022 |  |  |  |  |  |  |
| 1.384 |  | 0.017 |  |  |  |  |  |  |
| 0.44 |  | 0.016 |  |  |  |  |  |  |
| 0.25 |  | 0.026 |  |  |  |  |  |  |
| 0.561 |  | 0.016 |  |  |  |  |  |  |
| 0.984 |  | 0.03 |  |  |  |  |  |  |
| 0.43 |  | 0.065 |  |  |  |  |  |  |
| 1.534 |  | 0.014 |  |  |  |  |  |  |
| 1.37 |  | 0.026 |  |  |  |  |  |  |
| 0.911 |  | 0.135 |  |  |  |  |  |  |
| 3.793 |  | 0.104 |  |  |  |  |  |  |
| 0.027 |  | 0.018 |  |  |  |  |  |  |
| 0.024 |  | 0.053 |  |  |  |  |  |  |
| 0.083 |  | 0.055 |  |  |  |  |  |  |
| 0.047 |  | 0.033 |  |  |  |  |  |  |
| 0.048 |  | 0.054 |  |  |  |  |  |  |
| 0.031 |  | 0.122 |  |  |  |  |  |  |
| 0.031 |  | 0.018 |  |  |  |  |  |  |
| 0.065 |  | 0.031 |  |  |  |  |  |  |
| 0.035 |  | 0.042 |  |  |  |  |  |  |
| 0.026 |  | 0.051 |  |  |  |  |  |  |
| 0.038 |  | 0.076 |  |  |  |  |  |  |
| 0.036 |  | 0.011 |  |  |  |  |  |  |
| 0.029 |  | 0.016 |  |  |  |  |  |  |
| 0.057 |  | 0.181 |  |  |  |  |  |  |
| 0.065 |  | 0.041 |  |  |  |  |  |  |
| 0.063 |  | 0.258 |  |  |  |  |  |  |
| 0.078 |  | 0.013 |  |  |  |  |  |  |
| 0.031 |  | 0.07 |  |  |  |  |  |  |
| 0.059 |  | 0.036 |  |  |  |  |  |  |
| 0.424 |  | 0.058 |  |  |  |  |  |  |
| 0.051 |  | 0.066 |  |  |  |  |  |  |
| 0.014 |  | 0.022 |  |  |  |  |  |  |
| 0.031 |  | 0.02 |  |  |  |  |  |  |
| 0.118 |  | 0.028 |  |  |  |  |  |  |
| 0.092 |  | 0.036 |  |  |  |  |  |  |
| 0.094 |  | 0.047 |  |  |  |  |  |  |
| 0.124 |  | 0.048 |  |  |  |  |  |  |
| 0.056 |  | 0.121 |  |  |  |  |  |  |
| 0.075 |  | 0.053 |  |  |  |  |  |  |
| 0.06 |  | 0.059 |  |  |  |  |  |  |
| 0.025 |  | 0.054 |  |  |  |  |  |  |
| 0.038 |  | 0.027 |  |  |  |  |  |  |
| 0.061 |  | 0.067 |  |  |  |  |  |  |
| 0.06 |  | 0.042 |  |  |  |  |  |  |
| 0.026 |  |  |  |  |  |  |  |  |
| 0.056 |  |  |  |  |  |  |  |  |
| 0.031 |  |  |  |  |  |  |  |  |
| 0.04 |  |  |  |  |  |  |  |  |
| 0.014 |  |  |  |  |  |  |  |  |
| 0.084 |  |  |  |  |  |  |  |  |
| 0.025 |  |  |  |  |  |  |  |  |
| 0.013 |  |  |  |  |  |  |  |  |
| 0.024 |  |  |  |  |  |  |  |  |

* Samples from Close 01 extend into the lower rows of Close 03


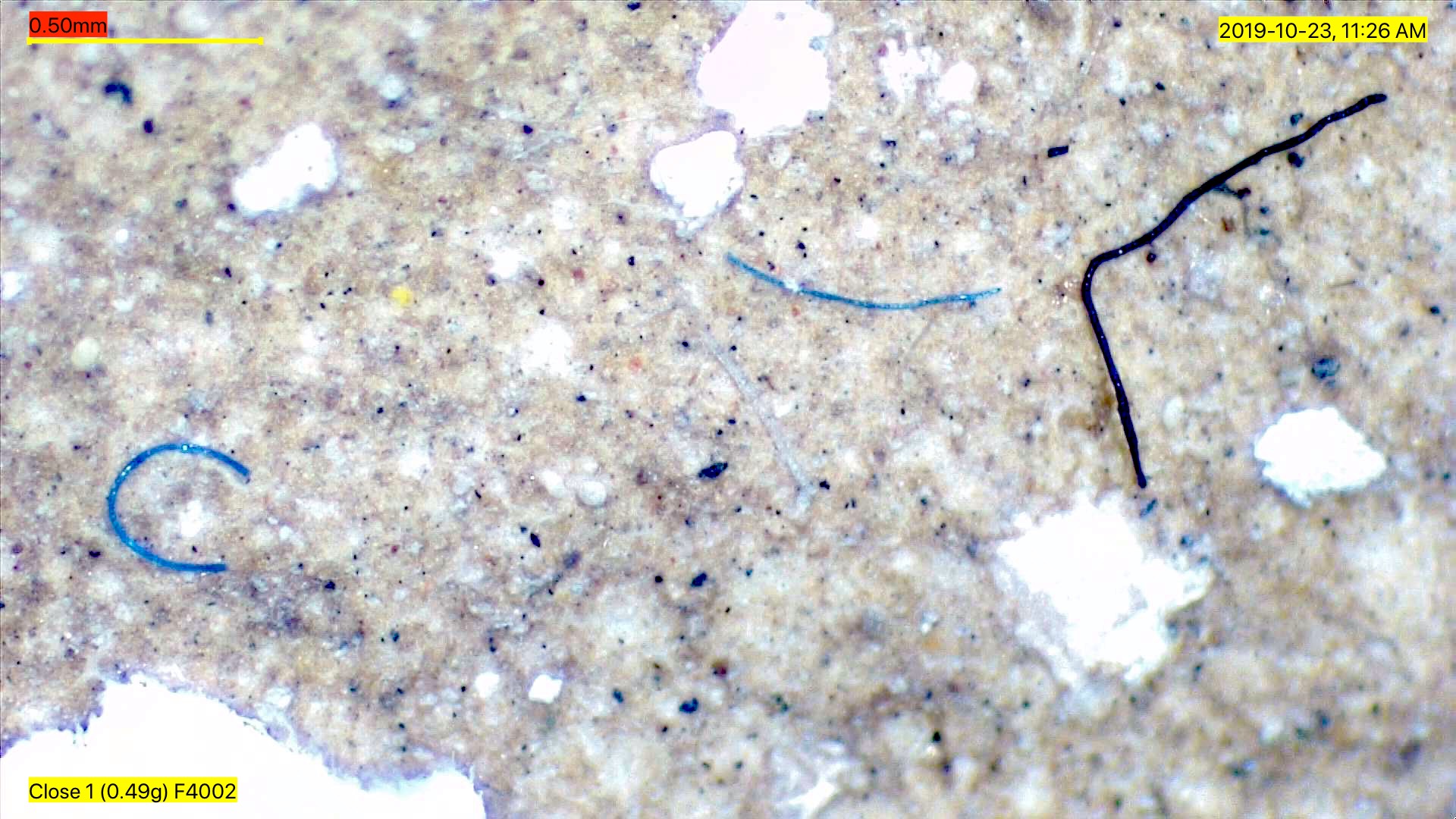

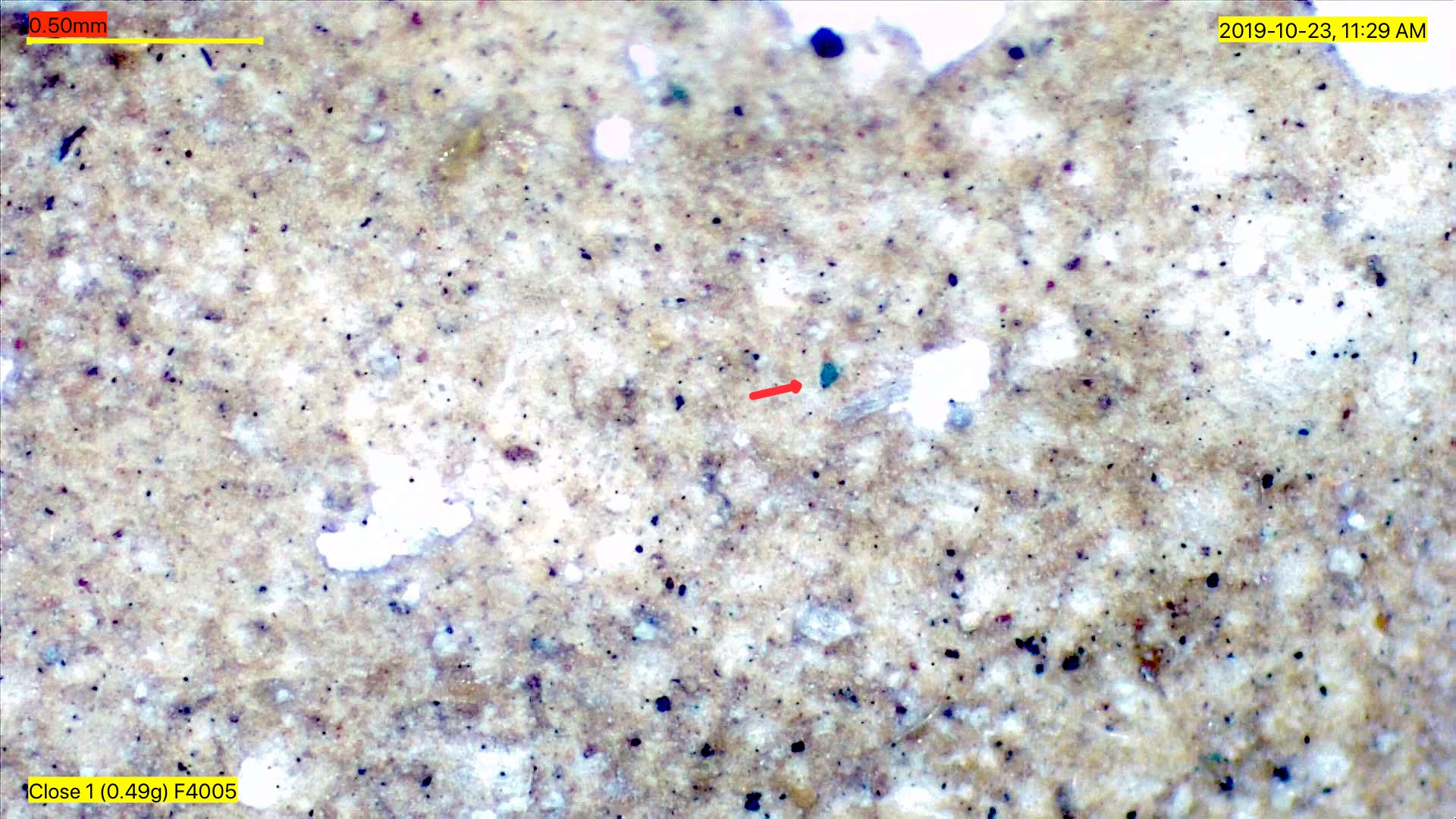


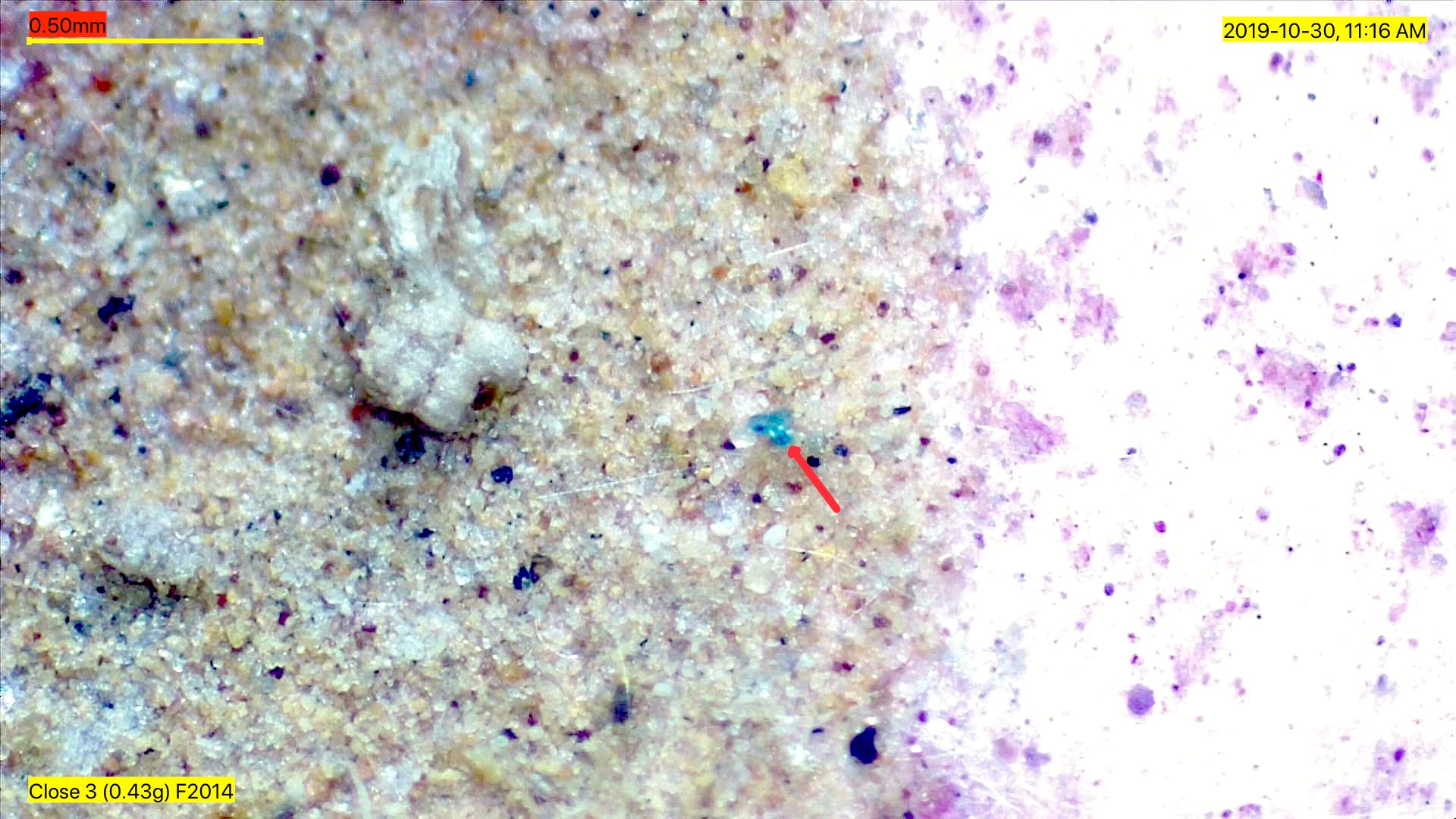


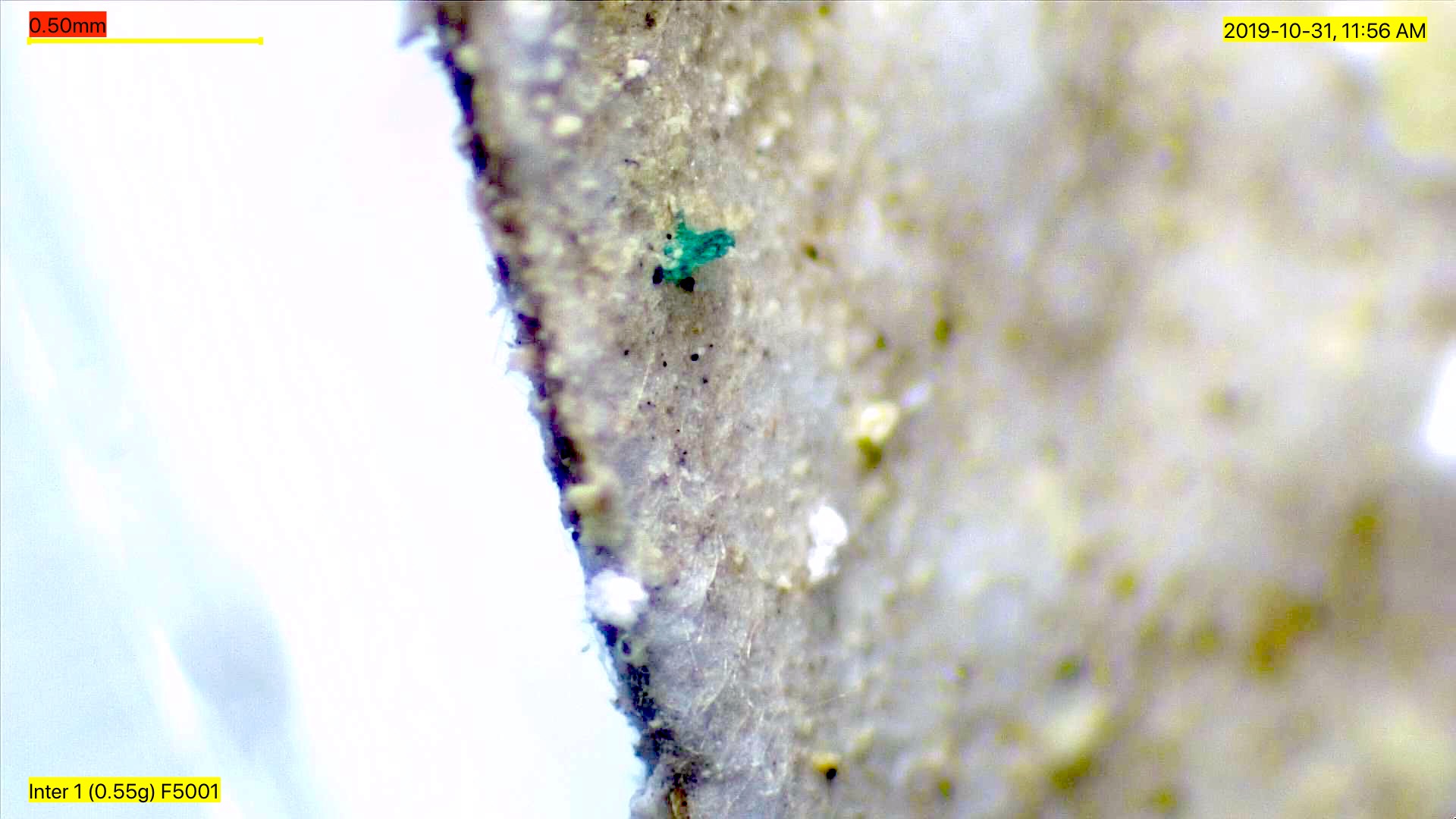


Figure SI-1. Photographs of example anthropogenic microfibres and fragments from the study sites (top to bottom) Close 01, Close 01, Close 03, and Intermediate 01.


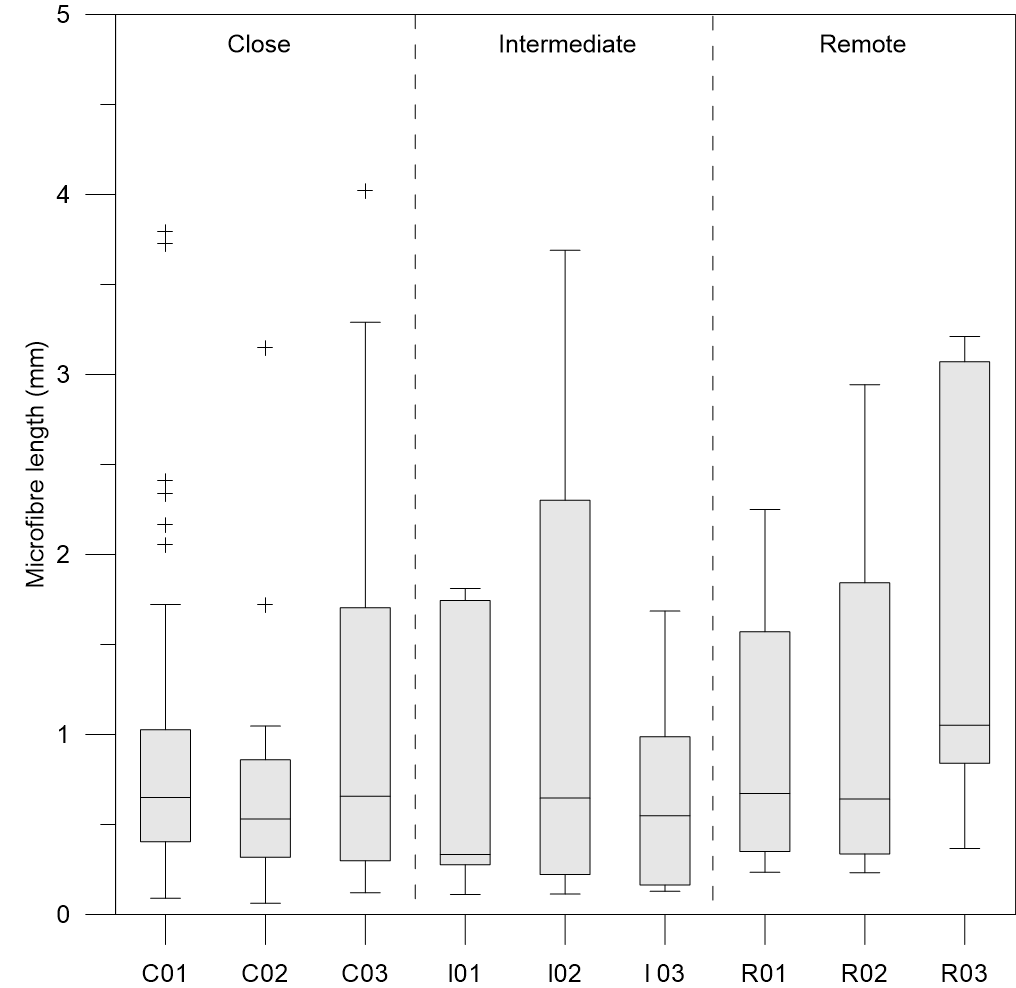

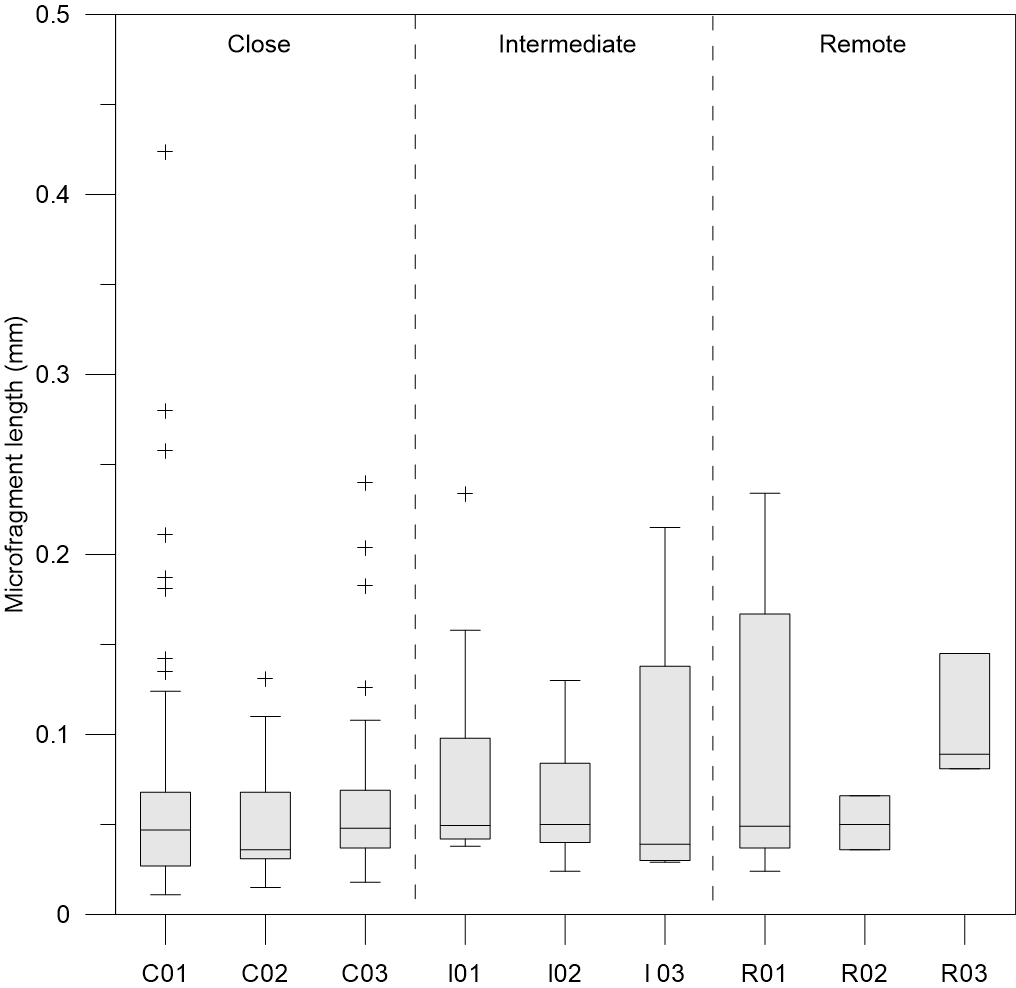


Figure SI-2. Distribution of microfibre (upper panel) and microfragment (lower panel) length (mm) at the three study sites (Close, Intermediate and Remote; each site has three replicates).


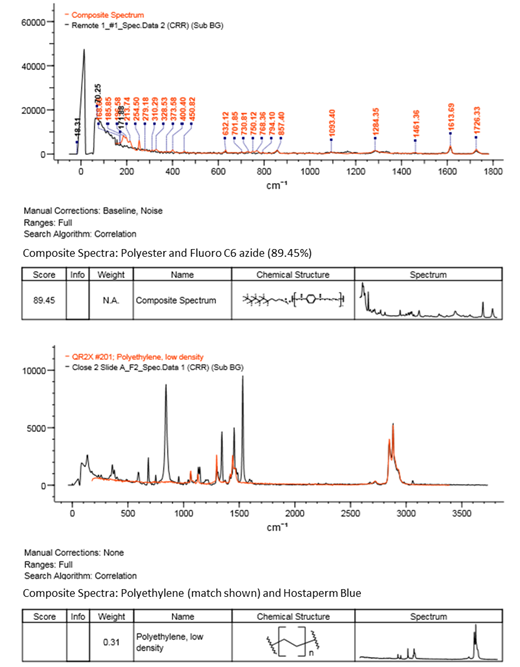


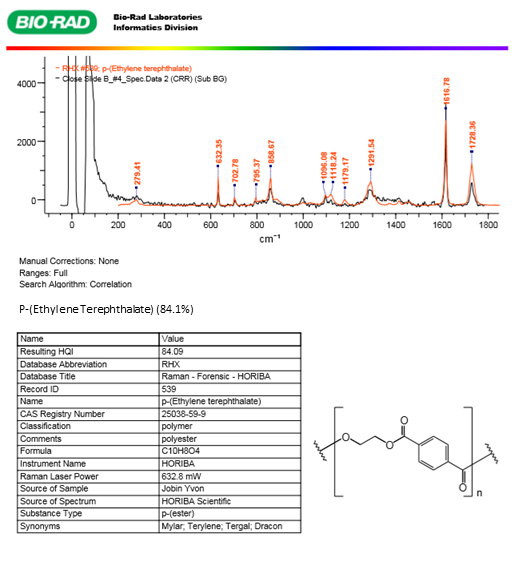


Figure SI-3. Raman spectral analysis reports from Bio-Rad KnowItAll online library for microfibres extracted from lichen samples at Remote 01 and Close 01, and a fragment from Close 02, showing matches with polyester, poly-terephthalate and polyetheylene. Raman spectroscopy measurements were carried out on a WiTech; fibres were analysed with a 785 nm laser using 50x-100x objectives and adjustable power (power ranged from 10 mW to 40 mW), and fragments were analysed with a 532 nm laser using 20x-50x objectives and adjustable power (power ranged from 15 mW to 20 mW). Raman spectra were recorded in the wavenumber range of 0-1,800 cm^-1^ with the 785 nm laser and 0-3,600 cm^-1^ with the 532 nm laser. The spectrum of the 50 particles were analysed through a commercial library (KnowItAll, Bio-Rad) to identify polymer type.
